# Procalcitonin kinetics in critically ill children: impact of continuous kidney replacement therapy modality and dose

**DOI:** 10.1007/s00467-026-07169-x

**Published:** 2026-02-11

**Authors:** Arife Ufacık Yöndem, Servet Yüce, Emrullah Aygüler, Ali Genco Gencay, Demet Demirkol

**Affiliations:** 1 Department of Pediatrics, Istanbul Faculty of Medicine, Istanbul University, Istanbul, Turkey; 2 Department of Public Health, Istanbul Provincial Health Directorate, Istanbul, Turkey; 3 Department of Pediatric Intensive Care, Istanbul Faculty of Medicine, Istanbul University, Millet Cad. Fındıkzade, Topkapı, Istanbul, 34093 Turkey

**Keywords:** Acute kidney injury, Critically ill children, Continuous kidney replacement therapy, Pediatric intensive care, Procalcitonin, Sepsis

## Abstract

**Background:**

Acute kidney injury (AKI) is common in critically ill children, frequently necessitating continuous kidney replacement therapy (CKRT). Procalcitonin (PCT) is widely used as an infection biomarker, yet its interpretation during CKRT remains unclear. Adult data regarding extracorporeal clearance of PCT are inconsistent, while pediatric evidence is limited.

**Methods:**

In this prospective observational study (May 2021–October 2023), 40 critically ill children receiving CKRT in a tertiary PICU were enrolled. Serum PCT was measured at CKRT initiation (T0), 12 h (T12), and 24 h (T24). CKRT modalities (CVVH, CVVHD, CVVHDF), effluent doses, and membrane types (PS, PAES, AN69-ST) were recorded. PCT kinetics were analyzed using non-parametric tests, with correlation assessed by Spearman’s rank.

**Results:**

Median baseline PCT was 3.6 ng/mL (IQR 0.5–27.2), rising to 7.4 (0.6–29.5) at T12 and stabilizing at 7.7 (0.6–30.5) at T24. Differences across time points were not statistically significant (*p* = 0.68). PCT trajectories were unaffected by CKRT modality, effluent dose, or membrane type, and no correlation was found between effluent dose and PCT changes. Stratification by high versus low effluent dosing revealed no significant differences. CKRT-related complications occurred in 17.5%, mainly filter clotting, without influencing PCT. PICU mortality was 35%, reflecting illness severity rather than CKRT.

**Conclusions:**

In pediatric CKRT, short-term PCT dynamics are driven by the underlying septic or inflammatory process rather than CKRT parameters. PCT typically peaks within 12 h of CKRT initiation and then stabilizes, supporting its reliability for infection monitoring and antibiotic stewardship during early CKRT. Larger studies are warranted to define long-term PCT behavior and prognostic utility.

**Graphical Abstract:**

**Figure:**
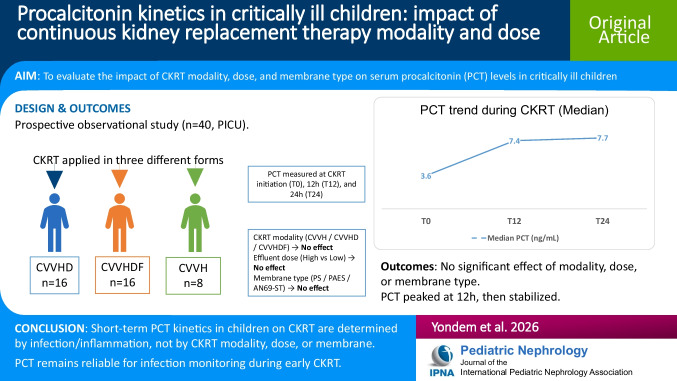
A higher resolution version of the Graphical abstract is available as Supplementary information.

**Supplementary Information:**

The online version contains supplementary material available at 10.1007/s00467-026-07169-x.

## Introduction

Acute kidney injury (AKI) is a frequent complication among critically ill children, affecting a considerable proportion of pediatric intensive care unit (PICU) patients and contributing substantially to morbidity and mortality [1–3]. Continuous kidney replacement therapy (CKRT) is widely employed in the PICU to manage severe AKI, fluid overload, refractory metabolic derangements, and sepsis-related multi-organ dysfunction [3–5]. Because many children requiring CKRT also present with sepsis and systemic inflammation, accurate monitoring of infection biomarkers in this population is of paramount clinical importance.

Procalcitonin (PCT) is a well-established biomarker for diagnosing and monitoring bacterial infections and sepsis in critically ill patients, including children [6, 7]. Elevated PCT concentrations can guide antimicrobial therapy and provide insight into the host inflammatory response [8, 9]. However, interpretation of PCT levels in patients receiving kidney support is complicated by the fact that both kidney dysfunction and extracorporeal therapies may influence PCT clearance. PCT is a 13-kDa peptide with a physiological half-life of approximately 20–24 h; under normal conditions, it is partially cleared by the kidneys, whereas kidney impairment leads to accumulation in the circulation [10–12]. Indeed, patients with acute or chronic kidney failure often exhibit higher baseline PCT values even in the absence of infection [13, 14]. Furthermore, CKRT may theoretically affect plasma PCT concentrations through convective removal or membrane adsorption. The actual impact of CKRT on PCT kinetics remains uncertain, as adult studies have produced conflicting results. Some reports indicate measurable clearance of PCT through CKRT circuits [15, 16], while others suggest minimal or no influence on circulating levels [17, 18]. Thus, it remains unclear whether elevated PCT reliably reflects infection in patients undergoing CKRT [19, 20].

Evidence regarding the interaction between CKRT and PCT in children is extremely limited. Previous studies have shown that pediatric patients on chronic hemodialysis often maintain persistently elevated PCT levels despite the absence of acute infection [12]. However, to date, no investigation has specifically addressed the short-term effects of different CKRT modalities or dosing strategies on PCT dynamics in critically ill children. Clarifying this relationship is clinically relevant, as PCT trajectories are increasingly used to guide antibiotic duration and escalation of care in septic pediatric patients, including those treated with CKRT.

In this prospective study, we sought to evaluate the influence of CKRT modalities, membrane types, and effluent doses on serum PCT concentrations in critically ill children. We hypothesized that early PCT trends during the first 24 h of CKRT would primarily reflect the underlying infectious or inflammatory state of the patient rather than technical differences in CKRT delivery.

## Materials and methods

### Study design, setting, and ethics approval and consent to participate

This prospective observational study was conducted in the multidisciplinary PICU of Istanbul Faculty of Medicine between May 2021 and October 2023. The study was approved by the Istanbul Faculty of Medicine Clinical Research Ethics Committee (approval no. 2021/811), and written informed consent was obtained from the parents or legal guardians of all participants.

### Study population

Critically ill pediatric patients requiring CKRT were screened for eligibility. Inclusion criteria were as follows: (i) age between 1 month and 18 years, (ii) presence of an indwelling central venous catheter suitable for CKRT, and (iii) planned CKRT duration of at least 24 h. Exclusion criteria included the following: age < 1 month or > 18 years, CKRT duration < 24 h (including patients who died or were transferred before completing 24 h), insufficient data availability, or lack of parental consent. Eligible patients were enrolled at the initiation of CKRT. No interventions beyond routine clinical care were performed; all CKRT prescriptions were determined by the attending physicians in accordance with unit protocols.

### CKRT indications and modality

Indications for CKRT included severe fluid overload, refractory metabolic acidosis, electrolyte disturbances, hyperammonemia or metabolic crisis, tumor lysis syndrome, AKI with oliguria or anuria, and sepsis-related multi-organ dysfunction unresponsive to conservative management. Three CKRT modalities were used: continuous venovenous hemofiltration (CVVH), continuous venovenous hemodialysis (CVVHD), and continuous venovenous hemodiafiltration (CVVHDF). All treatments were delivered with Prismaflex® (Baxter) or Fresenius® systems using appropriately sized filters. The modality choice was based on clinical needs and physician preference. We recorded each patient’s CKRT modality and the total duration of CKRT in hours.

### CKRT prescription and dose

Blood flow rates (mL/kg/min) and effluent flow rates were recorded at CKRT initiation (T0), 12 h (T12), and 24 h (T24). Anticoagulation was provided according to unit protocol, using either regional citrate anticoagulation or unfractionated heparin when not contraindicated. Membrane types were classified as polyethersulfone (PS), acrylonitrile 69 surface-treated (AN69-ST), or polyarylethersulfone (PAES).

### Data collection

Standardized case report forms were used to collect demographic, clinical, and laboratory data within the first 24 h of CKRT. Information included underlying diagnoses, primary reason for PICU admission, comorbidities, ventilatory and vasoactive support at CKRT initiation, and illness severity. Severity was assessed using the Pediatric Risk of Mortality III (PRISM-III) [21], Pediatric Logistic Organ Dysfunction-2 (PELOD-2) [22], and Pediatric Sequential Organ Failure Assessment (pSOFA) [23] scores. The vasoactive-inotropic score (VIS) [24] was calculated for patients receiving vasoactive agents. Fluid overload prior to CKRT was expressed as a percentage of body weight. Baseline kidney function was assessed using serum blood urea nitrogen (BUN), serum creatinine, and estimated creatinine clearance (Schwartz formula). Key laboratory results were documented at T0, T12, and T24.

### Procalcitonin measurements

Serum PCT—the primary biomarker of interest—was measured at T0, T12, and T24 hours. All samples were analyzed in the hospital’s central laboratory using an electrochemiluminescence immunoassay (Roche Cobas e411, Roche Diagnostics®), with results reported in ng/mL (lower detection limit, 0.02 ng/mL).

The first PCT sample (T0) was collected at the initiation of CKRT. For all patients, blood drawn for clinically indicated laboratory testing at CKRT initiation was used, and surplus material from these routine samples was utilized whenever available. When PCT was not part of the clinically ordered panel at a predefined time point, an additional sample was obtained concurrently with the next clinically required blood draw, ensuring that no extra invasive procedures were performed solely for research purposes.

Only patients who received CKRT for more than 24 h were included in the study. In addition to the predefined measurements within the first 24 h (T0, T12, T24), PCT values were also obtained every 24 h thereafter while CKRT continued. These extended measurements were not part of the original protocol but were collected as part of routine clinical monitoring.

For the purposes of the predefined study protocol and to ensure standardized, comparable time points across all patients, only the first 24-h measurements (T0, T12, T24) were included in the primary analysis. In addition to the predefined measurements obtained during the first 24 h (T0, T12, T24), serum PCT values were also available at approximately 24-h intervals thereafter while CKRT continued (up to a median of 108 h, with some patients monitored to 120 h). These longer-term measurements were evaluated in a secondary exploratory analysis to characterize PCT trends beyond the early phase of therapy. The results of this extended analysis are presented in the “Results” section and summarized both graphically and in Supplementary Table 1 and Figure 1.

### Outcomes and definitions

The primary outcome was the change in PCT level during the first 24 h of CKRT and whether this change differed according to modality, effluent dose, or membrane type. Secondary outcomes included PICU survival and complications related to CKRT.

### Statistical analysis

All analyses were performed using SPSS Statistics for Mac, version 28.0 (IBM Corp., Armonk, NY, USA). Continuous variables were summarized as medians with interquartile ranges (IQR), while categorical variables were expressed as counts and percentages. The distribution of continuous variables was assessed using the Kolmogorov–Smirnov test. Since PCT values were not normally distributed, non-parametric methods were applied. Changes in PCT levels across time points (T0, T12, T24) were examined with the Wilcoxon signed-rank test. Comparisons of PCT levels and their changes across CKRT modalities, membrane types, and anticoagulation strategies were performed using the Kruskal–Wallis test, with Mann–Whitney *U* tests applied for pairwise analyses when significant differences were detected. Patients were stratified into high- versus low-effluent groups according to the median effluent dose at each time point, and group comparisons of PCT changes were conducted with the Mann–Whitney *U* test. The association between effluent dose (as a continuous variable, mL/kg/h) and percent change in PCT levels (T12–T0, T24–T12, and T24–T0) was assessed using Spearman’s rank correlation. A two-tailed *p* value < 0.05 was considered statistically significant.

## Results

### Patient characteristics

A total of 40 pediatric patients fulfilled the inclusion criteria and were enrolled at the initiation of CKRT. Their demographic features, primary diagnoses, underlying conditions, and disease severity scores are summarized in Table 1. The most frequent indications for PICU admission were sepsis with multi-organ failure (42.5%), acute kidney injury (35%), acute encephalopathy (12.5%), and tumor lysis syndrome (10%). Prior to CKRT initiation, patients exhibited substantial kidney dysfunction and systemic inflammation, reflected in elevated serum creatinine, impaired creatinine clearance, and increased baseline PCT levels (Table 1).

**Table 1:** Demographic characteristics, diagnoses, and laboratory parameters of the study population

Variable	Data
**Age, months, median (IQR)**	45 (14–132)
**Sex, male, % (*n*)**	50 (20)
**PRISM III score, median (IQR)**	19 (15–25)
**PELOD II score, median (IQR)**	23 (13–42)
**pSOFA score, median (IQR)**	13 (10–17)
**Vasoactive–inotropic score (VIS), median (IQR)**	40.3 (12.5–185)
**Fluid overload, % body weight, median (IQR)**	4.8 (2.6–6.2)
**Primary diagnosis, % (*n*)**	
Sepsis and multiple organ failure	42.5 (17)
Acute kidney injury	35 (14)
Acute encephalopathy	12.5 (5)
Tumor lysis syndrome	10 (4)
**Underlying disease, % (*n*)**	
Inborn errors of metabolism	27.5 (11)
Malignancy	17.5 (7)
Chronic kidney disease	15 (6)
Chronic liver disease	10 (4)
Others	10 (4)
**Laboratory parameters at CKRT initiation**	
Serum BUN, mg/dL, median (IQR)	30.1 (8.3–46.3)
Serum creatinine (Cr), mg/dL, median (IQR)	1.34 (0.47–2.8)
Creatinine clearance (CrCl), mL/1.73 m^2^/min, median (IQR)	41.3 (20.3–106.8)
Procalcitonin (PCT), ng/mL, median (IQR)	3.62 (0.5–27.2)

*BUN* blood urea nitrogen, *Cr* creatinine, *CrCl* creatinine clearance, *PCT* procalcitonin, *PRISM* Pediatric Risk of Mortality, *PELOD* Pediatric Logistic Organ Dysfunction, *pSOFA* pediatric Sequential Organ Failure Assessment, *VIS* vasoactive–inotropic score, *IQR* interquartile range

### CKRT indications, modalities, and settings

Indications for CKRT were categorized into four groups, although overlapping conditions were common. Sixteen patients (40%) received CVVHD, 16 (40%) received CVVHDF, and 8 (20%) underwent CVVH. No patients changed modality during the 24-h observation period. Effluent and blood flow rates during CKRT are provided in Table 2.

**Table 2:** CKRT prescription parameters and procalcitonin (PCT) levels during the first 24 h

Variable	Data
Blood flow rates, mL/kg/min, median (IQR)	
T0	5.5 (3.0 − 7.9)
T12	5.0 (3.0 − 7.9)
T24	5.0 (3.0 − 7.4)
Effluent doses, m^2^/1.73/hours, median (IQR)	
T0	2910 (1963 − 4040)
T12	2208 (1853 − 4000)
T24	2100 (1922 − 3791)
Ultrafiltration rate, mL/kg/hours, median (IQR)	
T0	1.7 (0.5 − 2.0)
T12	1.9 (1.0 − 2.5)
T24	1.0 (0.5 − 2.2)
Procalcitonin (PCT), ng/mL, median (IQR)	
T0	3.6 (0.5 − 27.2)
T12	7.4 (0.6 − 29.5)
T24	7.7 (0.6 − 30.5)
Change in PCT Levels	
T12-T0 difference	− 0.01 (− 6.1 − 0.74)
T24-T12 difference	− 0.07 (− 1.18 − 0.07)
T24-T0 difference	− 0.08 (− 6.89 − 0.65)

*CKRT* continuous kidney replacement therapy, *PCT* procalcitonin, *IQR* interquartile range, *SD* standard deviation

Regarding membrane type, polysulfone (PS) was most frequently used (*n* = 27, 67.5%), followed by PAES (*n* = 8, 20%) and AN69-ST (*n* = 5, 12.5%). These proportions reflect stock availability and clinician preference. Regional citrate anticoagulation was employed in 19 patients (47.5%), unfractionated heparin in 11 (27.5%), and 6 patients (15%) were managed without anticoagulation; in 4 patients (10%), the anticoagulation strategy was changed during CKRT.

### Procalcitonin levels over 24 h of CKRT

At baseline, median PCT was markedly elevated (3.6 ng/mL, IQR 0.5–27.2), consistent with severe infection or systemic inflammation (Table 2). Median PCT values increased from T0 to T12 (7.4 ng/mL, IQR 0.6–29.5) and remained high at T24 (7.7 ng/mL, IQR 0.6–30.5). The Wilcoxon signed-rank test revealed no statistically significant changes between T12 and T0, T24 and T12, or T24 and T0 (*p* = 0.68), suggesting that CKRT did not significantly influence PCT clearance during the first 24 h. After an initial rise, PCT levels stabilized, with only minor, non-significant fluctuations between 12 and 24 h.

### Extended PCT measurements beyond 24 h (exploratory analysis)

In an exploratory post-hoc analysis, we evaluated PCT values obtained after the first 24 h of CKRT. Among 38 children with available measurements beyond 24 h, median PCT was 5.9 [0.6–29.5] ng/mL at 24 h and decreased to 1.7 [0.4–9.6] ng/mL at the last available measurement after 24 h (*p* < 0.001, Wilcoxon signed-rank test). The final PCT value was obtained at a median of 108 h (range 36–108 h) after CKRT initiation. These extended data suggest a gradual decline in PCT over time, although they were not part of the predefined primary analysis and should therefore be interpreted as exploratory (Supplementary Table 1 and Supplementary Figure 1).

### Comparison by effluent dose

Spearman’s correlation analysis showed no significant association between effluent dose and PCT levels at either T12 or T24 nor between effluent dose and changes in PCT (T12–T0, T24–T12, T24–T0) (all *p* > 0.05, Table 3). When analyzing the changes, no correlation was found between the change in PCT levels and the change in effluent dose at T12–T0, T24–T12, and T24–T0 (*p* > 0.05, Table 3). Essentially, increasing the volume of blood purified per hour did not result in a greater decrease in PCT during the first day of therapy.

**Table 3:** Correlation between effluent dose and procalcitonin (PCT) levels and changes during the first 24 h

Effluent dose		PCT levels			PCT difference		
		T0	T12	T24	T12 − T0	T24 − T12	T24 − T0
**T0**	*r*	NA	− 0.058	− 0.096	0.082	− 0.107	0.026
	*p*		0.729	0.566	0.627	0.523	0.877
**T12**	*r*	− 0.140	− 0.048	− 0.085	0.119	− 0.103	0.061
	*p*	0.396	0.772	0.607	0.471	0.535	0.713
**T24**	*r*	− 0.164	0.006	− 0.038	0.206	− 0.106	0.136
	*p*	0.313	0.973	0.815	0.202	0.516	0.401

The correlations between effluent dose (continuous variable, mL/kg/h) and PCT levels or changes were assessed using Spearman’s rank correlation coefficient (*r*)

*CKRT* continuous kidney replacement therapy, *PCT* procalcitonin, *NA* non-applicable

Patients stratified into high- versus low-effluent groups demonstrated similar PCT levels at all time points. Although baseline PCT appeared higher in the low-effluent group, the difference was not statistically significant (Table 4). Overall, categorizing by effluent volume did not significantly affect PCT levels or changes, indicating that effluent volume does not strongly correlate with the inflammatory response or clinical course as measured by PCT.

**Table 4:** Procalcitonin (PCT) levels and changes according to effluent dose groups

PCT, ng/mL, median (IQR)	Effluent dose, median (IQR)								
	T0 < Median	T0 ≥ median	***p***-value	T12 < median	T2 ≥ median	***p***-value	T24 < median	T24 ≥ median	***p***-value
**T0**	12.5 (0.8–38.1)	1.6 (0.3–24.3)	0.12	12.5 (0.8–38.1)	2.0 (0.3–20.8)	0.20	4.2 (0.6–38.1)	2.0 (0.5–17.3)	0.42
**T12**	9.8 (2.7–27.7)	6.2 (0.4–35.9)	0.47	8.7 (2.7–27.7)	8.2 (0.4–33.6)	0.57	5.6 (0.6–27.7)	8.7 (0.5–31.2)	0.99
**T24**	9.7 (2.3–30.7)	8.4 (0.4–32.7)	0.41	8.6 (2.3–30.7)	6.6 (0.5–30.8)	0.48	4.9 (0.5–30.7)	8.4 (0.6–28.8)	0.88
**PCT difference**									
**T12-T0**	− 0.3 (− 11.9–0.8)	0.0 (− 1.2–5.0)	0.34	− 0.3 (− 11.9–0.8)	0.0 (− 0.9–3.6)	0.30	0.0 (−11.9–0.6)	0.0 (− 2.0–5.0)	0.58
**T24-T12**	− 0.2 (− 1.3–0.0)	0.0 (− 2.4–0.4)	0.62	− 0.1 (− 0.9–2.4)	0.0 (− 2.6–0.1)	0.90	− 0.1 (− 0.5–0.1)	− 0.1 (− 2.4–0.1)	0.51
**T24-T0**	− 0.4 (− 8.9–2.8)	0.0 (− 3.5–0.7)	0.40	− 0.4 (− 8.9–7.4)	0.0 (− 2.5–0.7)	0.34	− 0.2 (− 8.9–0.7)	0.0 (− 3.5–0.7)	0*.*63

All group comparisons were performed using the Mann–Whitney *U* test for two-group analyses. A two-tailed *p* < 0.05 was considered statistically significant

*CKRT* continuous kidney replacement therapy, *PCT* procalcitonin, *IQR* interquartile range

### Comparison by CKRT modality

Median baseline PCT values were comparable across the three modalities. PCT increased until T12 and remained elevated at T24 in all groups. Neither absolute PCT levels nor changes over time differed significantly among patients treated with CVVH, CVVHD, or CVVHDF (Table 5). Thus, CKRT modality did not exert a measurable effect on short-term PCT kinetics.

**Table 5:** Association between CKRT modality and procalcitonin (PCT) levels

PCT, ng/mL, median (IQR)	Modality			*p*-value
	CVVHD (***n*** = 16)	CVVH (***n*** = 8)	CVVHDF (***n*** = 16)	
T0	3.05 (0.42 − 26.22)	20.58 (2.69 − 68.14)	3.11 (0.76 − 16.88)	0.280
T12	4.44 (0.42 − 33.03)	11.63 (2.21 − 26.03)	9.24 (1.05 − 22.1)	0.812
T24	3.02 (0.39 − 39.05)	8.36 (1.89 − 30.2)	8.5 (1 − 16.53)	0.779
**PCT difference**				
T12–T0	0.01 (− 7.65 − 0.08)	0.6 (− 22.08 − 0.49)	0 (− 2.14 − 5.79)	0.593
T12–T24	− 0.04 (− 0.71 − 2.94)	− 0.32 (− 0.89 − − 0.03)	− 0.08 (− 2.03 − 0.23)	0.669
T24–T0	− 0.05 (− 5.86 − 8.82)	− 0.96 (− 20.28 − − 0.13)	0.02 (− 4.51 − 1.74)	0.304

Comparisons of PCT levels and changes between CKRT modalities were performed using the Kruskal–Wallis test. A two-tailed *p* < 0.05 was considered statistically significant

*CKRT* continuous kidney replacement therapy, *CVVHD* continuous venovenous hemodialysis, *CVVH* continuous venovenous hemofiltration, *CVVHDF* continuous venovenous hemodiafiltration, *PCT* procalcitonin, *IQR* interquartile range

Baseline PCT values showed some variation across CKRT modalities and membrane types, with higher T0 levels observed in children who received CVVH and in those treated with AN69-ST membranes. These subgroups included proportionally more patients with severe sepsis and higher inflammatory burden at CKRT initiation, which likely explains their elevated baseline PCT levels. However, these differences were not statistically significant, and PCT trajectories over time did not diverge between groups, indicating that the observed baseline variation reflected underlying clinical heterogeneity rather than modality- or membrane-related effects.

### Comparison by membrane type

Similarly, PCT levels at T0, T12, and T24 did not differ significantly across patients treated with AN69-ST, PAES, or PS membranes. No membrane type demonstrated superior clearance capacity for PCT (Table 6).

**Table 6:** Association between membrane type and procalcitonin (PCT) levels

PCT, ng/mL, median (IQR)	Membrane type			p-value
	AN69-ST (***n*** = 5)	PAES (***n*** = 8)	PS (***n*** = 27)	
T0	21.7 (1.24 − 99.9)	8.33 (1.47 − 22.75)	2.9 (0.47 − 24.27)	0.694
T12	9.82 (6.19 − 30.2)	6.72 (1.55 − 16.81)	5.58 (0.59 − 31.2)	0.765
T24	9.67 (8.6 − 29.7)	5.92 (1.35 − 14.6)	3.62 (0.54 − 41.8)	0.737
**PCT difference**				
T12–T0	− 11.88 (− 60.9 − − 0.02)	− 1.56 (− 6.09 − 0.09)	0.01 (− 1.18 − 9.22)	0.129
T24–T12	− 0.15 (− 0.5 − 0.38)	− 0.2 (− 1.1 − 0.03)	− 0.06 (− 1.28 − 0.05)	0.944
T24–T0	− 12.03 (− 63.6 − 0.36)	− 2.82 (− 7.39 − − 0.05)	0 (− 1.43 − 11.02)	0.167

PCT levels and changes between membrane types were performed using the Kruskal–Wallis test. A two-tailed *p* < 0.05 was considered statistically significant

*CKRT* continuous kidney replacement therapy, *PCT* procalcitonin, *PS* polysulfone, *PAES* polyarylethersulfone, *AN69-ST* acrylonitrile 69 surface-treated, *IQR* interquartile range

### Adverse events

CKRT-related complications occurred in 7 patients (17.5%) within 24 h. The most common event was circuit clotting requiring unplanned filter replacement (*n* = 5). One patient developed transient ionized hypocalcemia related to citrate anticoagulation, which was corrected with calcium infusion and conversion to heparin. Another patient had minor catheter-site bleeding on heparin, which resolved without discontinuation of CKRT. None of these events had a direct impact on PCT levels.

### Mortality

The overall PICU mortality rate was 35% (14/40). Non-survivors typically presented with higher illness severity scores and refractory septic shock. Among survivors, the median PICU length of stay was 16 days (range 1–90). All deaths were attributable to the severity of underlying illness and multi-organ failure; no mortality was directly related to CKRT complications.

## Discussion

In this prospective study, we examined whether CKRT prescription parameters—modality, effluent dose, and membrane type—alter early PCT kinetics in critically ill children. During the first 24 h of treatment, none of these CKRT variables significantly affected circulating PCT concentrations. Instead, PCT dynamics were largely determined by the underlying septic or inflammatory response, with levels typically peaking at approximately 12 h and thereafter stabilizing. These observations indicate that short-term CKRT does not obscure PCT trends in pediatric practice.

Our findings stand in contrast to several reports in adult populations demonstrating substantial PCT clearance during extracorporeal therapy. Dahaba et al. evaluated 13 ICU patients with sepsis-induced MODS undergoing CVVH and observed a notable decrease in efferent plasma PCT concentrations, attributed largely to membrane adsorption [18]. Similarly, Herget-Rosenthal and colleagues reported an 83% reduction in PCT across 377 patients with varying stages of chronic kidney disease undergoing either intermittent hemodialysis (IHD) or peritoneal dialysis [13]. Mori et al. investigated 76 adult patients undergoing 4-h IHD sessions with high-flux membranes and found that serum PCT levels decreased by approximately 19% following dialysis [25]. Similarly, Caldini et al. demonstrated a dramatic reduction in serum PCT levels—ranging from 51 to 89% over the observation period—with high-cut-off PAES-type hemofilters [8]. Based on these findings, they cautioned that PCT should be interpreted with particular care in patients receiving high-cut-off CKRT, as extracorporeal clearance may confound its reliability as an infection biomarker [8].

In a Japanese multicenter prospective study, PCT levels were assessed in 123 adult patients undergoing a single 4-h IHD session, irrespective of infection status. High-flux membranes were consistently used, and a 46% reduction in PCT was observed following IHD. The investigators emphasized that this decline may vary depending on membrane type and recommended that pre-dialysis PCT levels be considered in clinical decision-making for this population [16]. Kade et al. investigated the effect of CVVHD with PAES membranes in 36 patients with septic shock and AKI [9]. After 24 h of therapy, they reported a 50% decrease in serum PCT, attributed to both anti-inflammatory effects and transmembrane clearance. Zhang et al. [19] evaluated 60 adult patients with sepsis-induced AKI treated with either IHD or CKRT. The greatest decline occurred within the first 72 h after KRT initiation, with both groups showing a rapid early decrease followed by a more gradual decline through day 7. Notably, the magnitude of PCT reduction did not differ significantly between intermittent and continuous modalities, suggesting that both forms of KRT exert similar effects on PCT kinetics in adults.

A prospective single-blind randomized controlled trial from Korea investigated PCT elimination in 24 patients undergoing CVVH with PAES membranes. An immediate decline in PCT levels was detected at the onset of treatment, likely reflecting membrane adsorption. However, by the conclusion of the study, the investigators determined that CKRT had no significant effect on overall PCT concentrations [26]. Similarly, Meisner et al. studied 26 adult patients with sepsis-related kidney failure treated with CVVHF. Although PCT was clearly removed from plasma during the procedure, this did not translate into altered circulating levels. The authors concluded that PCT elimination depends on the duration of therapy and the degree of membrane adsorption, but that PCT remains a reliable biomarker even in patients with AKI undergoing CVVHF [17]. In a more recent study, Level et al. assessed 13 critically ill adults undergoing CVVH with either AN69 or polyamide membranes; approximately 20% of PCT was cleared across the membrane, primarily through convective clearance and adsorption during the early hours of treatment. Nevertheless, this partial clearance did not result in significant changes in systemic PCT levels [7].

In contrast to these predominantly adult studies, our findings in a pediatric cohort suggest that CKRT does not significantly affect serum PCT levels in the short term. Despite differences in modalities (CVVH, CVVHD, CVVHDF), effluent doses, and membrane types, PCT kinetics during the first 24 h of therapy were largely unchanged. Rather than declining due to extracorporeal clearance, PCT concentrations tended to rise initially and then stabilize, reflecting ongoing systemic production in response to infection and inflammation. This observation highlights a fundamental difference between pediatric and adult populations: while extracorporeal therapy may substantially influence PCT dynamics in adults, in critically ill children, the biomarker appears to remain robust and interpretable during CKRT, at least in the early phase of treatment. Multiple explanations likely account for this discrepancy. First, pediatric patients exhibit distinct inflammatory dynamics compared with adults, with higher endogenous PCT production during early sepsis that may exceed extracorporeal clearance. Second, standard pediatric CKRT membranes (PS, PAES, AN69-ST) generally exhibit lower adsorption capacity than high-cut-off filters used in some adult cohorts, thereby limiting measurable removal. Third, continuous modalities may result in slower, more gradual solute clearance compared with the rapid concentration shifts observed in IHD. Finally, our analysis focused exclusively on the early 24-h period, during which ongoing inflammatory production may overshadow any membrane-related elimination. These combined factors likely explain why PCT kinetics in our pediatric cohort remained stable despite active CKRT.

Another key observation from our cohort is that PCT trajectories did not differ between hemofiltration and hemodialysis techniques, nor between higher- and lower-effluent-dose groups. Evidence regarding the influence of CKRT modality on PCT remains limited. In a prospective observational study, Mouche et al. examined 40 pediatric patients across three centers with kidney failure and found that PCT levels decreased by 75% with hemodiafiltration and by 35% with hemodialysis in patients without infection. However, after three dialysis sessions within 1 week, no sustained reduction compared to baseline was observed [12]. Similarly, in our analysis, increasing the effluent dose did not yield measurable differences in PCT kinetics. Correlation testing confirmed the absence of a linear association between effluent dose and PCT change, indicating that more “intensive” CKRT dosing is unlikely to artificially suppress PCT concentrations or, conversely, cause accumulation at lower doses. This finding is clinically important, as it reassures clinicians that PCT trends can be interpreted consistently across a broad spectrum of standard CKRT prescriptions.

The impact of membrane type on PCT removal has been another major focus of research. Montagnana et al. demonstrated that membrane properties can significantly affect post-dialysis PCT values: high-flux membranes generally reduce PCT more effectively than low-flux membranes, whereas less permeable membranes may retain higher levels [20]. In our study, patients were treated with PS, PAES, or AN69-ST membranes; however, no significant differences in PCT kinetics were observed among these groups. None of these membranes appeared superior in removing PCT in vivo. It should be noted, however, that we did not employ specialized “high cut-off” filters, which are not commonly used in pediatric practice. Based on the pediatric membranes available, our data indicate that membrane type does not significantly affect short-term PCT clearance.

## Limitations and strengths

Our study has several limitations. First, the sample size was relatively small, limiting the statistical power to detect subtle differences between subgroups such as membrane type or CKRT modality. Second, the analysis was confined to the first 24 h of therapy; longer-term effects of CKRT on PCT kinetics were not assessed. Over extended treatment periods, cumulative extracorporeal clearance might have a more pronounced impact. Third, despite standardized measurement times, natural variability in PCT trajectories among septic patients could have introduced noise—some patients may have been on an upward trend while others were plateauing, independent of CKRT. Fourth, we lacked a comparator group of critically ill children with sepsis who did not undergo CKRT, which would have clarified whether observed PCT dynamics were attributable to the therapy or to the natural course of sepsis. Such a design is difficult to achieve in practice due to ethical and logistical considerations, as withholding CKRT when indicated is not feasible. Finally, we did not measure PCT directly in the effluent fluid, which could have provided quantitative insights into extracorporeal removal.

In an exploratory post-hoc analysis, we also evaluated PCT values obtained beyond the first 24 h of CKRT in a subset of patients. These extended measurements demonstrated a gradual decline in PCT over the subsequent days, consistent with both resolution of inflammation and the cumulative effects of ongoing extracorporeal support. However, because these measurements were available only in a limited number of patients and were not part of the predefined primary analysis, we report them as exploratory. Therefore, our main conclusions remain limited to the early phase of CKRT, and longer-term PCT kinetics should be investigated in larger, prospective studies.

Despite these limitations, our study contributes novel evidence in an underexplored area. To our knowledge, this is the first prospective analysis examining the impact of different CKRT prescriptions on PCT kinetics in critically ill children. The prospective design, consistent measurement strategy, and standardized CKRT protocols enhance the validity of our findings. Moreover, the inclusion of a heterogeneous PICU population increases the applicability of our results to real-world clinical practice, where CKRT is used across a wide range of diagnoses and illness severities.

## Clinical implications

An important concern in clinical practice is whether CKRT might artificially lower PCT levels and mask a rising trend, thereby complicating antibiotic stewardship decisions. Our findings provide reassurance in the pediatric setting: short-term PCT trajectories remained stable and interpretable regardless of CKRT modality or effluent dose. This observation is consistent with the study by Ceccarelli et al., who demonstrated in septic shock patients receiving CKRT and ECMO that PCT-guided antibiotic therapy remained feasible and reliable [27]. Importantly, in our cohort, persistent PCT elevation after 24 h of CKRT in a child who showed no clinical improvement most likely reflected ongoing infection or inflammation rather than insufficient dialysis. Conversely, a decline in PCT accompanied by clinical recovery can be regarded as a trustworthy marker of improvement, since CKRT is unlikely to artificially reduce PCT to misleading levels. Taken together, these findings support the continued use of PCT as a valuable tool for infection monitoring and antibiotic stewardship in critically ill children undergoing CKRT.

## Future directions

Further research in larger pediatric cohorts is needed to validate these results and to characterize PCT kinetics beyond the first 24 h of CKRT. Examining longer-term trajectories and correlating PCT clearance with clinical outcomes such as organ recovery and survival will provide valuable insights. Moreover, assessing other biomarkers alongside PCT—such as C-reactive protein or emerging markers like presepsin—may help determine whether certain biomarkers are more reliable or less affected by extracorporeal therapies [28, 29]. Such investigations could refine the use of infection biomarkers in critically ill children requiring CKRT.

## Conclusion

In critically ill children receiving CKRT, serum PCT concentrations during the first 24 h appear to be driven primarily by the underlying septic or inflammatory state rather than by CKRT modality, effluent dose, or membrane type. PCT typically peaks around 12 h after CKRT initiation and then stabilizes, indicating that standard CKRT prescriptions do not substantially alter short-term PCT kinetics. These findings suggest that PCT remains interpretable during the early phase of CKRT, with persistent elevations more likely reflecting ongoing inflammation than extracorporeal removal. Nonetheless, larger studies with extended follow-up are needed to confirm these observations and to better characterize longer-term PCT behavior during CKRT.

## Supplementary Information

Below is the link to the electronic supplementary material.

ESM 1 Graphical abstract (PPTX 84.3 KB)

ESM 2 (DOCX 22.0 KB)

## Data Availability

The datasets generated and/or analyzed during the current study are not publicly available due to patient privacy and institutional regulations but are available from the corresponding author upon reasonable request.
