# Supplementary material for: Procalcitonin kinetics in critically ill children: impact of continuous kidney replacement therapy modality and dose
Source: Pediatr Nephrol. 2026 Feb 11;41(7):2229–38. doi: 10.1007/s00467-026-07169-x (PMC13197258; doi:10.1007/s00467-026-07169-x)
Supplement: Supplementary file 1 — Graphical abstract (PPTX 84.3 KB) [file 467_2026_7169_MOESM1_ESM.pptx]

## Slide 1
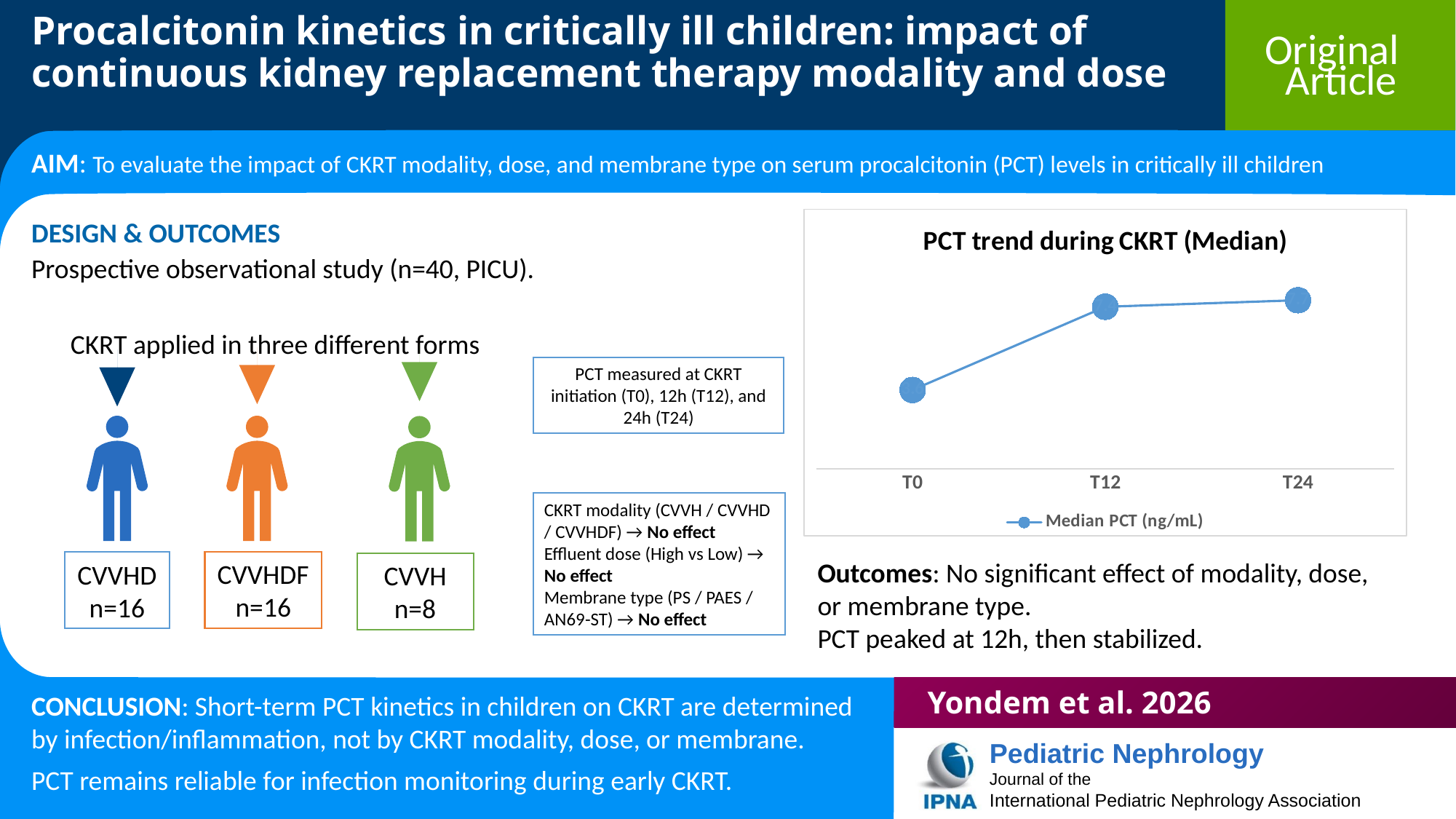

Procalcitonin kinetics in critically ill children: impact of continuous kidney replacement therapy modality and dose
AIM: To evaluate the impact of CKRT modality, dose, and membrane type on serum procalcitonin (PCT) levels in critically ill children
### Chart: PCT trend during CKRT (Median)
| Category | Median PCT (ng/mL) |
|---|---|
| T0 | 3.6 |
| T12 | 7.4 |
| T24 | 7.7 |DESIGN & OUTCOMES
Prospective observational study (n=40, PICU).
CKRT applied in three different forms
PCT measured at CKRT initiation (T0), 12h (T12), and 24h (T24)
CKRT modality (CVVH / CVVHD / CVVHDF) → No effect
Effluent dose (High vs Low) → No effect
Membrane type (PS / PAES / AN69-ST) → No effect
Outcomes: No significant effect of modality, dose, or membrane type.
PCT peaked at 12h, then stabilized.
CVVHDF n=16
CVVHD n=16
CVVH n=8
Yondem et al. 2026
CONCLUSION: Short-term PCT kinetics in children on CKRT are determined by infection/inflammation, not by CKRT modality, dose, or membrane.
PCT remains reliable for infection monitoring during early CKRT.
