# Supplementary material for: Procalcitonin kinetics in critically ill children: impact of continuous kidney replacement therapy modality and dose
Source: Pediatr Nephrol. 2026 Feb 11;41(7):2229–38. doi: 10.1007/s00467-026-07169-x (PMC13197258; doi:10.1007/s00467-026-07169-x)
Supplement: Supplementary file 2 — (DOCX 22.0 KB) [file 467_2026_7169_MOESM2_ESM.docx]

## **Supplementary Table 1. Procalcitonin (PCT) values beyond 24 hours in children receiving CKRT (exploratory analysis)**

| **Time interval (hour)** | **Median PCT (ng/mL)** | **IQR (ng/mL)** |
| --- | --- | --- |
| **24 h** | **5.92** | 0.55 – 29.48 |
| **24–48 h** | **5.22** | 0.80 – 14.90 |
| **48–72 h** | **3.13** | 0.72 – 10.00 |
| **72–96 h** | **1.89** | 0.60 – 7.85 |
| **96–120 h** | **1.55** | 0.55 – 4.85 |

**Supplementary Figure 1.** Median PCT levels measured at 24 h and at subsequent intervals (24–48 h, 48–72 h, 72–96 h, and 96–120 h) in children receiving CKRT. Values represent medians for all available measurements at each time point. This exploratory analysis demonstrates a gradual decline in PCT beyond the first 24 h of therapy.
